# Coronary artery-bronchial artery fistulas: report of two Dutch cases with a review of the literature

**DOI:** 10.1007/s12471-014-0518-z

**Published:** 2014-01-26

**Authors:** S. A. M. Said, R. M. Oortman, J.-H. Hofstra, P. M. J. Verhorst, R. H. J. A. Slart, M. W. de Haan, F. Eerens, H. J. G. M. Crijns

**Affiliations:** 1 Department of Cardiology, Hospital Group Twente, Geerdinksweg 141, 7555 DL Hengelo, the Netherlands; 2 Department of Cardiology, Thoraxcentrum Twente, Medisch Spectrum Twente, 7513 ER Enschede, the Netherlands; 3 Department of Cardiology, Koningin Beatrix Hospital, 7101 BN Winterswijk, the Netherlands; 4 Department of Nuclear Medicine and Molecular Imaging, University Medical Center Groningen, 9713 GZ Groningen, the Netherlands; 5 Department of Radiology, Maastricht University Medical Center, 6229 HX Maastricht, the Netherlands; 6 Department of Cardiology, Maastricht University Medical Center, 6229 HX Maastricht, the Netherlands

**Keywords:** Congenital anomaly, Coronary bronchial artery fistulas, Multi-detector computer tomography, Positron emission tomography/^13^-ammonia-adenosine scanning, Management

## Abstract

**Background:**

Coronary bronchial artery fistulas (CBFs) are rare anomalies, which may be isolated or associated with other disorders.

**Materials and methods:**

Two adult patients with CBFs are described and a PubMed search was performed using the keywords “coronary bronchial artery fistulas” in the period from 2008 to 2013.

**Results:**

Twenty-seven reviewed subjects resulting in a total of 31 fistulas were collected. Asymptomatic presentation was reported in 5 subjects (19 %), chest pain (*n* = 17) was frequently present followed by haemoptysis (*n* = 7) and dyspnoea (*n* = 5). Concomitant disorders were bronchiectasis (44 %), diabetes (33 %) and hypertension (28 %). Multimodality and single-modality diagnostic strategies were applied in 56 % and 44 %, respectively. The origin of the CBFs was the left circumflex artery in 61 %, the right coronary artery in 36 % and the left anterior descending artery in 3 %. Management was conservative (22 %), surgical ligation (11 %), percutaneous transcatheter embolisation (30 %), awaiting lung transplantation (7 %) or not reported (30 %).

**Conclusions:**

CBFs may remain clinically silent, or present with chest pain or haemoptysis. CBFs are commonly associated with bronchiectasis and usually require a multimodality approach to be diagnosed. Several treatment strategies are available. This report presents two adult cases with CBFs and a review of the literature.

## Introduction

Coronary bronchial artery fistulas (CBFs) are usually found incidentally during invasive coronary angiography (CAG) [1]. The aetiology of CBFs is uncertain. CBFs are often associated with bronchiectasis which may be bilateral [1] or unilateral either to the left [2] or to the right lung [3]. Sometimes CBFs occur concomitant with tetralogy of Fallot, supravalvular aortic stenosis or Takayasu aortitis [4]. The morphology may be shown using several diagnostic modalities: CAG, magnetic resonance imaging (MRI) and multi-detector computed tomography (MDCT). MDCT identified the course of CBFs between the circumflex artery (Cx) and the bronchial arteries [2, 5] and myocardial perfusion imaging (MPI) revealed reversible defects [5]. The functional assessment may be obtained by MPI using technetium-99 m tetrofosmin, MRI and oximetric series during cardiac catheterisation to establish the magnitude of the shunt. CBFs often remain asymptomatic but they can also be the source of aggravating haemoptysis [6]. CBFs can be managed either by a conservative medical regimen, percutaneous occlusion techniques or surgical ligation.

We report two adult patients with angina pectoris in whom CAG demonstrated significant obstructive coronary artery disease (CAD), and coincidentally CBFs were found. The first patient was treated with stenting of the Cx coronary artery, and in a second session the fistulous vessel was occluded by coiling during a percutaneous transcatheter embolisation (PTE) procedure. The second patient had associated bronchiectasis, sustained a subclinical anterior wall myocardial infarction (MI), and was treated medically for his CBF. The literature is briefly reviewed.

## Methods

Two adult patients with CBFs are presented and a PubMed search was performed using the keywords “coronary bronchial artery fistulas” in the period between 2008 and 2013. This search harvested 30 citations. Eighteen relevant papers were selected and evaluated yielding 27 patients with 31 CBFs.

## Case reports

1. A 66-year-old female patient with known arterial hypertension and diaphragmatic hernia was evaluated for palpitations, persistent typical and atypical chest pain which has been present for 3 years. In 2008, the patient underwent stenting of the Cx for a significant lesion. Physical examination was unremarkable except for a body mass index of 27 kg/m^2^. A 12-lead ECG was normal and the results of a bicycle exercise tolerance test were equivocal. Ambulatory ECG recording depicted normal rhythm variations. Echocardiography showed normokinetic biventricular function and normal valvular function with an ejection fraction of 60 %. The myocardial perfusion test showed a fixed perfusion defect in the inferior and apical region without reversibility (Fig. 1a). Cardiac MRI imaging was normal without evidence of delayed enhancement after gadolinium. She had neither haemoptysis nor a pulmonary disorder. Conventional CAG demonstrated a patent stent in the Cx without other abnormalities. The myocardial fractional flow reserve for the right coronary artery (RCA) was 0.94, proximal left (PL) Cx-II 0.92 and PLCx-I 0.86. A suspicion was raised suggesting a fistula between the proximal part of the RCA and the right bronchial artery (Fig. 1b). MDCT documented a fistulous connection between the proximal branch of the RCA and the right bronchial artery.

**Fig. 1:**
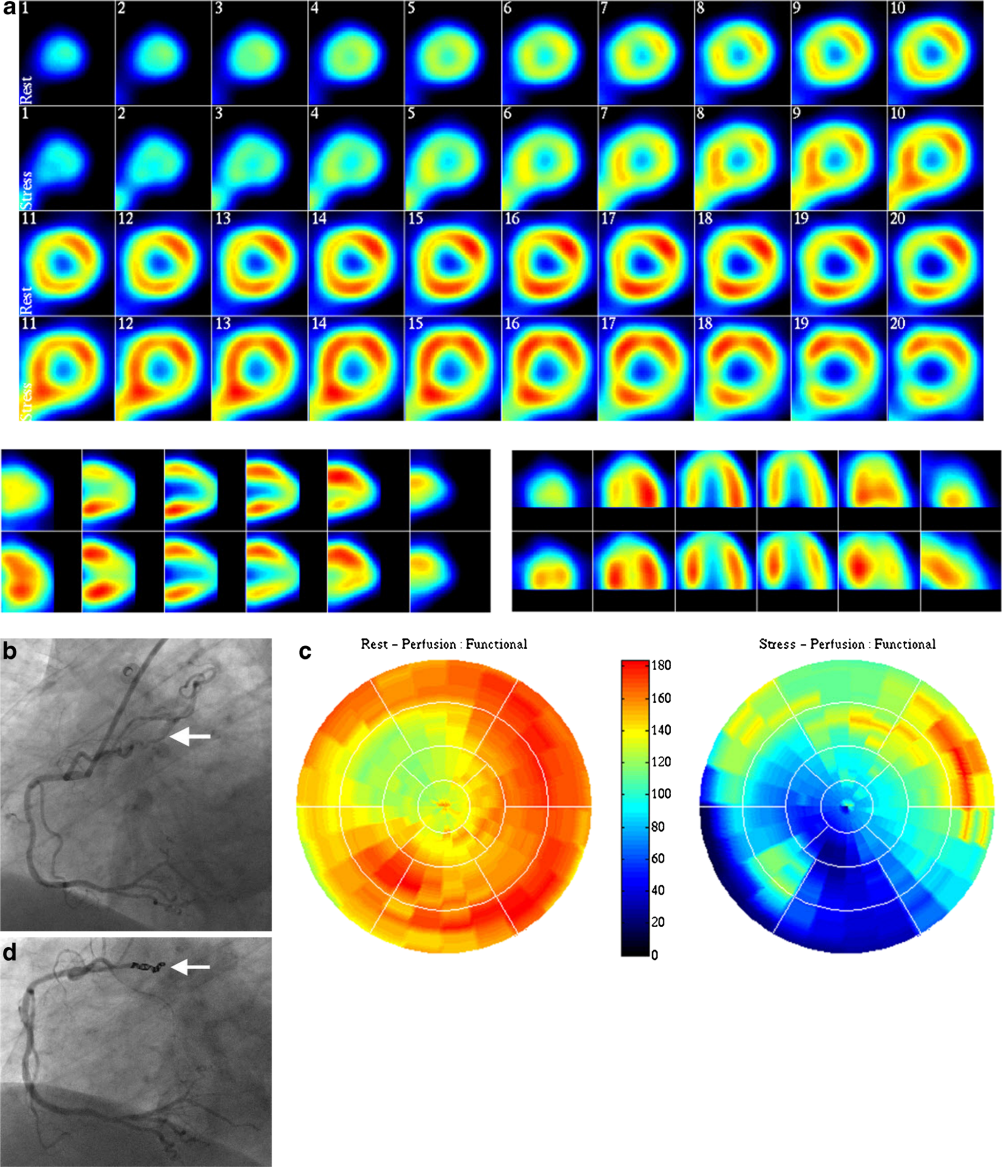
**a** Myocardial perfusion imaging (MPI) demonstrating the irreversible defect of the inferior segment, **b** Coronary angiographic frame of RCA demonstrating a CBF between a proximal branch of RCA and bronchial artery before coiling (*arrow*), **c** Normal findings on the rest ^13^N-ammonia polar map (left panel) and a large absolute perfusion defect (*dark blue*) in the inferior wall after pharmacological stress with adenosine (*right panel*) and **d** Coronary angiographic frame of RCA demonstrating disappearance of the CBF between a proximal branch of RCA and bronchial artery after coiling (*arrow*)
  Gated adenosine stress/rest ^13^N-ammonia PET/CT visually demonstrated an apical left ventricular (LV) defect at rest which increased during adenosine stress reaching the basal inferior and inferolateral regions (Fig. 1c). The global stress/rest ratio was 0.74 with a high resting flow. The regional stress/rest ratio was LAD 0.81, RCA 0.41 and Cx 0.85. Blood flow through the left anterior descending artery (LAD) and Cx arteries was twofold higher than through the RCA. The RCA was the fistula donor vessel visible on angiography. Normal LV function without stunning was noticed on gated PET. A severe decrease of the segmental perfusion reserve was detected in the basal inferior and basal inferolateral areas. Based on the findings of PET-CT scanning, evidence was delivered for decision-making to perform an intervention. Percutaneous occlusion of the fistulous vessel was achieved with the application of 4 coils. After coiling of the CBF (Fig. 1d), the procedure was complicated by distal embolisation to the RCA and right ventricular (RV) branch by a thrombus formed at the tip of the micro-perfusion catheter, through which the coils were placed in the fistula, which dislodged on retrieval of the micro-catheter at the end of the procedure. This gave rise to chest pain, ST elevation in the inferior leads, intermittent second-degree AV block and cardiogenic shock. She was treated with 7500 IU of intravenous heparin, and an intracoronary bolus of abciximab accompanied by thrombosuction. The flow in the RCA was restored with a thrombolysis in myocardial infarction (TIMI) flow score of 3. Her haemodynamic condition stabilised further following the administration of atropine 1 mg intravenously and fluid expansion. After full recovery, follow-up trans-thoracic echocardiography (TTE) revealed normal LV systolic function without wall motion disturbances. During follow-up (now over 2 years) she has remained free from chest pain while treated with acetylsalicylic acid, omeprazole and perindopril.
2. A 74-year-old male patient with hypertension, chronic obstructive pulmonary disease and bronchiectasis was evaluated because of angina pectoris due to subacute anterior wall MI. The clinical examination was unremarkable except for a blood pressure of 90/54 mmHg and pulse rate of 92 beats/min. The ECG on admission showed a normal regular sinus rhythm at 94 beats/min, QS in V1-V3 and persistent ST elevation in V1-V6 without reciprocal depression compatible with a semi-recent anterior wall myocardial infarction. The chest X-ray revealed bilateral basal bronchiectasis (Fig. 2a). Echocardiography revealed akinesia of the anterior segment with moderate LV kinetics and without significant valvular dysfunction.

**Fig. 2:**
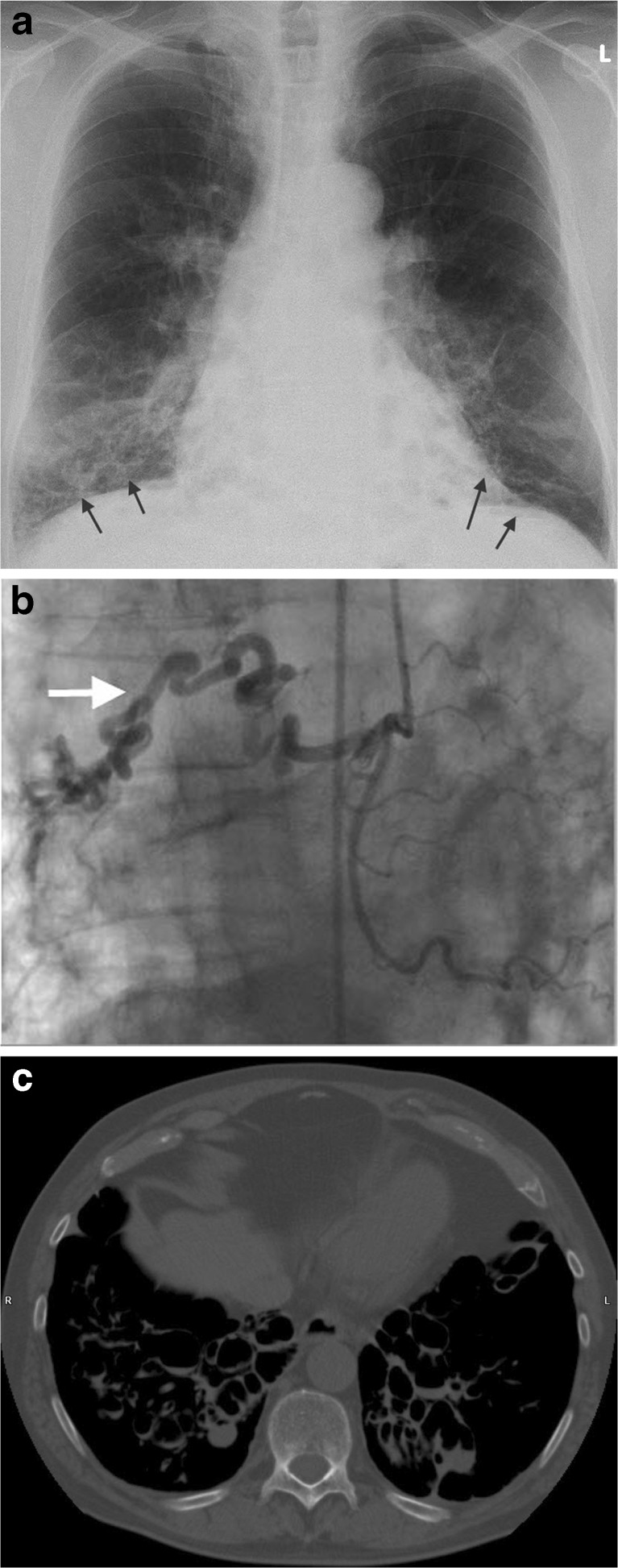
**a** Chest X-ray demonstrating bronchiectasis at the right and left lower regions of the lung (*arrows*), **b** Coronary angiographic frame of RCA in AP position, depicting the CBF between a proximal branch of the RCA and bronchial artery (*arrow*) and **c** Thoracic computed tomography scan showing the bilateral bronchiectasis
  Conventional CAG demonstrated one-vessel disease with a subtotal stenosis in the proximal LAD and fistulous multiple tiny vessels exiting from a proximal branch of the small calibre RCA with a possible connection to the right pulmonary artery and right bronchial artery (Fig. 2b). At cardiac catheterisation mild pulmonary hypertension (capillary wedge pressure 16, RV pressure 53/9 and pulmonary artery pressure 40/16 mmHg), normal cardiac output (6.0 ml/min) and on oximetric series no left-to-right shunt was found. MDCT confirmed bilateral cystic bronchiectasis of the basal pulmonary fields and left middle lobe (Fig. 2c). The LAD showed calcification with suspected high-grade stenosis, a normal Cx and RCA. Furthermore, a CBF from the proximal part of the RCA communicated with the right bronchial artery. Radionuclide shunt measurement and MPI demonstrated a fixed defect in the anterior and apical region without reversibility with an LV ejection fraction of 48 % without a detectable left-right shunt. Pulmonary ventilation/perfusion scintigraphy revealed bilateral matched wedge-shaped lesions in the basal lung segments excluding pulmonary embolism. Cardiovascular magnetic resonance investigation (CMR) showed bilateral pulmonary lesions and no viable myocardium of the anterior wall with moderate LV dysfunction. PCI was abandoned and medical management was instituted. He was treated with metoprolol, acetylsalicylic acid, perindopril, simvastatin, tiotropium, acetylcysteine, and formoterol/beclometasone. He remains free of symptoms and was scheduled for annual follow-up.

## Review subjects

Eighteen papers were selected yielding 27 patients. The mean age was 60 years (range 29–81). There were 8 females and 19 males (Table 1). Two patients had bilateral and another had multilateral fistulas from all three coronary arteries. A total of 31 CBFs were detected. The majority were unilateral (*n* = 24). The origin of the fistula was the Cx in 19 (61 %), the RCA in 11 (36 %) and the LAD in 1 (3 %) of the fistulas. Asymptomatic presentations occurred in 5 (19 %) subjects, chest pain (*n* = 17), haemoptysis (*n* = 7), dyspnoea (*n* = 5), history of pulmonary tuberculosis (*n* = 3) and cystic fibrosis (*n* = 2). The applied diagnostic modalities, single or combined, were MDCT (22/27; 81 %), conventional CAG (18/27; 67 %), MPI and cardiac MRI (4/27; 15 %) and TTE (8/27; 30 %). Associated CAD defined as the number of vessel disease (VD) with significant stenosis was present in 33 % and subclassified as follows: 1VD (*n* = 3), 2VD (*n* = 3), 3VD (*n* = 1) and significant left main (LM) disease (*n* = 2). Concomitant disorders were bronchiectasis (*n* = 12; 44 %), arterial hypertension (*n* = 7; 26 %) and diabetes mellitus (*n* = 9; 33 %). Management was conservative medical strategy (*n* = 6; 22 %), awaiting lung transplantation (*n* = 2; 7 %), surgical ligation (*n* = 3; 11 %) combined with coronary artery bypass grafting (*n* = 2) or pulmonary lobectomy (*n* = 1), PTE (*n* = 8; 30 %, including one failure), and was not reported (*n* = 8; 30 %). Materials used, alone or combined, for PTE were microcoils (*n* = 2), coils (*n* = 3) and polyvinyl particles (*n* = 3).

**Table 1:** Literature review from 2008 to 2013

Author/reference	Age/gender/clinical presentation	Diagnostic modalities	CBA fistula	Associated disorders	Management
Kang et al [5]. 2008	67F/CP	MPI/MDCT/CAG	Cx-left BA	Bronchiectasis left lower lobe	Conservative
Lee et al [12]. 2008	67F/CP	MDCT	Cx	Bronchiectasis RR	Right middle lobe and both lower lobes
	69M/CP		RCA	2VD	
	56F/CP		Cx	DM 3VD	
	53M/asymptomatic		RCA	1VD RR hypercholesterolaemia	
	45M/asymptomatic		Cx	DM RR	
	49F/CP		Cx	1VD	
	61F/asymptomatic		Cx	DM stroke	
	76M/CP		Cx	2VD	
Khalpey et al [25]. 2009	29M/screening/end-stage lung disease/cystic fibrosis	CAG	Cx-right BA	Bronchiectasis/cystic fibrosis/inflammatory phlegmon	Lung transplant
Bas et al [36]. 2010	64M/anginal chest pain	CAG/MDCT	RCA-left BA	LM stenosis	CABG + SL
Rigattieri et al [27]. 2010	78M/ACS	CAG/MDCT/TTE	RCA-BA	2VD	PCI conservative
Bury et al [1]. 2010	62F/anginal chest pain	MRI/MDCT/CAG	Cx-BA + PA	Anomalous left PA and anomalous Cx from proximal RCA bilateral basal bronchiectasis	Conservative
Forouzandeh et al [26]. 2011	40M/screening/cystic fibrosis/	CAG/MDCT	LAD Cx RCA-BA	Cystic fibrosis	Pre-lung transplant
Shin et al [2].2011	54M/chest tightness/haemoptysis	MDCT	Cx-BA	DM RR LAD 50 % stenosis bronchiectasis left lung	PTE microcoils + polyvinyl alcohol particles
Kul et al [43]. 2011	60M/chronic haemoptysis/chest pain	TTE/chest CT/CAG	RCA-left lung	1VD LAD stenosis	PCI LAD PTE coils
Song [7] 2011	51M/chest pain/palpitation	MDCT/CAG/TTE/ETT/Holter	Cx-BA right	DM SVT	Conservative
Kim [9] 2011	35M/chest pain	MPI/MDCT/CAG	RCA, Cx-BA	Bronchiectasis	PTE coils
Lee et al [22]. 2012	57M/massive haemoptysis/dyspnoea/chest pain	MDCT/TTE/CAG	RCA-BA	DM bronchiectasis Left lower lung field	Surgery, left lower lung lobectomy + removal of CBF fistula
Yoon et al [6]. 2012	70M/massive haemoptysis	CAG	Cx-left BA	Pulmonary tuberculosis/CHF/arrhythmia/thoracic spondylitis	PTE microcoils + PVA particles
Yoon et al [6]. 2012	57M/cardiac arrest/haemoptysis	CT/CAG	RCA-BA	DM/pneumonia/pulmonary tuberculosis	PTE failed
Yoon et al [6]. 2012	68F/haemoptysis	CAG	RCA, Cx-BA left lung	Pulmonary tuberculosis bronchiectasis left lung	PTE gelfoam + PVA particles
Ybarra et al [3]. 2012	62M/anginal chest pain/dyspnoea	CT/MPI/TTE/MDCT/CAG	Cx-BA right	DM/RR bronchiectasis chronic bronchitis/interstitial fibrosis	PTE coils
Nacer et al. [44] 2012	81M/chest pain/dyspnoea	TTE/CAG	RCA-BA right	COPD/bronchiectasis LM stem stenosis	CABG + SL
Parida et al [40]. 2013	75M/chest pain/haemoptysis/dyspnoea/syncope	TTE/CAG/CT	Cx-BA right	RR/bronchiectasis right lung	PTE covered stent
Galli et al [45]. 2013	71F/chest pain	TTE/CAG/CT	Cx-BA left	DM RR hypercholesterolaemia	Conservative
Eryilmaz et al [46]. 2013	64M/dyspnoea on exertion/	CAG/MDCT	Cx-BA right	bronchiectasis	Conservative, refusal of surgery
Case 1	66F/chest pain	CAG/MDCT/MPI/PET-CT	RCA-BA	1VD	PTE/PCI Cx
Case 2	74M/chest pain	CAG/PV scan/MDCT/MRI	RCA-BA right	1VD/COPD/bronchiectasis	Conservative

*ACS* acute coronary syndrome, *BA* bronchial artery, *CABG* coronary artery bypass grafting, *CAG* coronary angiography, *CBF* coronary bronchial fistula, *CHF* congestive heart failure, *COPD* chronic obstructive pulmonary disease, *CP* chest pain, *CT* computed tomography, *Cx* circumflex coronary artery, *DM* diabetes mellitus, *LAD* left anterior descending coronary artery, *LM* left main stem, *MDCT* multi-detector computed tomography, *MPI* myocardial perfusion imaging, *MRI* magnetic resonance imaging, *PA* pulmonary artery, *PCI* percutaneous coronary intervention, *PTE* percutaneous transcatheter embolization, *PVA* polyvinyl alcohol particles, *RCA* right coronary artery, *RR* hypertension, *SL* surgical ligation, *SVT* supraventricular tachycardia, *TTE* transthoracic echocardiography, *VD* vessel disease

## Discussion

### History and incidence

CBFs may have a unilateral [7, 8] or bilateral [9] presentation. As early as 1803, a Cx coronary artery to right bronchial artery communication was initially described by von Haller [10]. In 1972, Smith et al. presented the first angiographic case of a unilateral CBF [11]. The MDCT and conventional CAG incidence of CBFs is estimated at 0.61 % (8/1300) [12] and 0.5 % [4], respectively.

### Embryology

Small, not functional anastomoses between the bronchial arteries and the coronary arteries exist. These anastomoses have been regarded as congenital in origin. Coronary angiographic visualisation was demonstrated by Bjork in 1966; 22 % of normal subjects had such anastomoses and it was found in 48 % of patients with obstructive CAD [13]. However, these arterial communications may become enlarged and functional in a variety of cardiovascular entities including pulmonary artery hypoplasia, tetralogy of Fallot, supravalvular aortic stenosis and Takayasu arteritis and may be associated with pulmonary disorders such as pulmonary thromboembolism [4, 14–16]. CBFs are probably already present at birth and remain clinically silent in most cases. These congenital vascular communications are usually small in size and haemodynamically insignificant. The factors regulating the existence or re-opening and growth of these vascular anastomoses are as follows. First: disequilibrium of the pressure gradient between the coronary, bronchial and pulmonary vascular trees may lead to growth of CBFs giving rise to increased flow from the coronary to the bronchial artery vascular bed [17]. Shunting of blood from the coronary to the bronchial circulation occurs either when the coronary artery pressure increases as in supravalvular aortic stenosis [18], or when bronchial artery pressure decreases as in pulmonary atresia and tetralogy of Fallot. Second: in obstructive CAD, the bronchial-to-coronary artery fistula has been demonstrated to fill the distal coronary vascular bed distal to the proximal obstruction [19, 20]. And finally, myocardial ischaemia [9] or MI after transbronchial artery embolisation of bronchial-to-pulmonary vascular fistula for management of haemoptysis have been reported [21].

### Symptomatology and associated disorders

The clinical features of patients with CBFs are diverse and the severity often depends on the magnitude of the shunt and concomitant disorders. Although chest pain and/or dyspnoea related to steal phenomenon [12] is the most common symptom of CBFs, massive and sometimes fatal haemoptysis may occur [2, 6, 22]. Haemoptysis was found in 26 % of the reviewed subjects. In 2003, Jim et al., reported haemoptysis in 17 % of their reviewed subjects. It has been postulated that persistent infection and inflammation of the bronchial wall results in vasodilatation which causes lowering of the resistance in the bronchial vascular bed and stimulates the fistula growth [23]. Localised bronchiectasis is the most common entity associated with CBFs [23, 24] but pulmonary tuberculosis, cystic fibrosis, chronic bronchitis and interstitial fibrosis have also been reported [3, 6, 25, 26]. Importantly, in patients on the pulmonary ward with persisting haemoptysis after embolisation of the bronchial artery and undocumented coronary anatomy, coronary imaging has to be performed to exclude the possibility of a CBF. Here we report two adult patients presenting with angina pectoris who were found to have CAD and coincidentally detected CBFs during conventional CAG and confirmed by MDCT. Our first patient did not have associated pulmonary disease, the second patient did have bilateral basal bronchiectasis. Neither had haemoptysis. In our review, bronchiectasis was found in 44 % followed by CAD in 33 % of subjects. Jim et al., in their review of 12 reported cases in 2003, found that the most common associations were bronchiectasis (67 %) followed by CAD (33 %) [23].

### Diagnosis

The diagnosis and functional assessment of CBFs are challenging. Currently CBFs are readily diagnosed by non-invasive imaging methods such as MDCT [1, 27]. MDCT is considered the diagnostic procedure of choice in patients with coronary artery anomalies in whom conventional CAG may result in misinterpretation or inability to identify multiple fistulas, or the course and termination site of the fistula [28]. A multimodality diagnostic approach is frequently applied [7–9]. This approach was applied in both our patients. In this current review, in slightly less than half (44 %) of the reviewed subjects, single-modality diagnostic strategies were used and multimodality diagnostic approaches were applied in 56 %, to establish the diagnosis and assess the functional characteristics of CBFs. In the majority of subjects the origin of CBF was from the LCA followed by the RCA in 65 % and 35 %, respectively. These data are consistent with previously published report by Lee et al.: 75 % and 25 %, respectively [12]. Jim et al., in their review of 12 reported cases in 2003, found that the CBFs originated from the Cx in 83 % of cases [23].

Positron emission tomography/^13^N-ammonia-adenosine scanning has been applied to assess the functional status and flow quantification in CAD [29, 30] which could also be applied in CBFs. In our first patient a myocardial perfusion PET-CT study with^13^N-ammonia at rest and during adenosine pharmacological stress was performed, which showed extensive ischaemia in the basal inferior and inferolateral regions with preserved LV function. Quantitative PET data showed impaired regional coronary flow reserve (<2.0) in the RCA territory, thus allowing a precise and reliable evaluation of the irreversible myocardial perfusion defect (Fig. 1a). Based on these findings percutaneous coil embolisation was advocated.

Currently, coronary artery fistulas with vascular termination (e.g., pulmonary artery) [31] or cameral communication (e.g., the left ventricle) [32] may be noninvasively identified and assessed with MDCT. MDCT identified the morphology and the course of CBF between the Cx and left bronchial arteries and myocardial perfusion scintigraphy provided its functional assessment [5]. The morphology may be shown using several diagnostic modalities. Both patients underwent selective contrast CAG, myocardial perfusion test and MDCT for symptoms suspected of myocardial ischaemia.

The functional assessment may be obtained with technetium-99 m tetrofosmin, MRI and oximetric series during cardiac catheterisation to establish the magnitude of the shunt. Positron emission tomography/^13^N-ammonia-adenosine scanning has been applied to investigate the functional status and absolute flow quantification (ml/min/g) in CAD [29, 30] and can also be used in CBFs. The use of ^13^N-ammonia PET-CT for functional assessment of coronary artery fistulas in adults may yield additional diagnostic information not obtained with technetium-99 m tetrofosmin scintigraphy. In adult subjects, ^13^N-ammonia-adenosine PET-CT myocardial scanning has proven to be valuable for the functional assessment of CAD [30, 33] and of congenital coronary artery fistulas [34]. Echocardiogram has a pivotal role in cardiac function monitoring in clinical practice. TTE was performed in our two patients to assess cardiac function and exclude other disorders. Although TTE is essential to evaluate associated cardiac anomalies, it was performed only in one-third (30 %) of the reviewed subjects.

### Therapy

Although, in the majority of cases, CBFs remain silent, the fistula should be closed in symptomatic patients. For CBFs in asymptomatic pre-transplant patients with end-stage lung disease, PTE is not indicated [25]. Occlusion of CBFs in symptomatic patients is achieved by surgical ligation [35, 36], or PTE using a detachable balloon [37, 38], microspheres [38], coils [3, 38], microcoils [2], polyvinyl alcohol [6] or a covered stent [39, 40]. In 1983, Reidy et al. described the first case successfully treated with a percutaneous occlusion technique applying a detachable balloon in a male patient with CBFs [37]. In our first case, the PTE procedure was complicated by MI due to distal thrombus embolisation into the main coronary artery branch, which was aborted after rapid and adequate intervention. MI has been reported as a serious complication of bronchial artery embolisation [21]. To enhance the occlusion of the fistula with the percutaneous embolisation process during coiling no anticoagulants were used. We believe that a fine balance should be kept in anticoagulant use to potentiate thrombus formation and simultaneously minimise the risk of inadvertent embolisation. MI is a rare complication of percutaneous embolisation of CBFs. In the series of Jama et al. (*n* = 29), they described their experience of percutaneous occlusion of coronary artery fistulas and reported complications in four patients (14 %) [41]. Coronary occlusive thrombosis of the main artery occurred in one, device migration in two and coronary spasm in one patient [41]. As an alternative to surgery, bronchial artery embolisation is an established treatment modality for patients with haemoptysis [19, 35]. In some selected cases, a thorcoscopic approach for surgical ligation of CBFs, when technically amenable, may be considered an alternative to the percutaneous procedure [42]. In cases of severe CAD, coronary artery bypass graft with ligation of the fistula may be a better treatment.

## Abbreviations

AP: Anteroposterior
CBF: Coronary-bronchial artery fistula
RCA: Right coronary artery
